# Mowing management favors primary productivity and carbon sequestration without changing species diversity in a temperate hayfield in Central Interior British Columbia, Canada

**DOI:** 10.1371/journal.pone.0317536

**Published:** 2025-03-18

**Authors:** Sarah Bayliff, Wendy Gardner, Jay Prakash Singh, Lauchlan Fraser

**Affiliations:** Thompson Rivers University, Kamloops British Columbia, Canada; University of Ferrara, ITALY

## Abstract

We examined the effects of different mowing heights on the plant and soil characteristics of an irrigated and fertilized perennial cropping system in the central interior of British Columbia, Canada primarily composed of *Medicago sativa*, *Phleum pratense*, and *Trifolium pratense*. Mowing treatments included cutting heights of 0 cm, 5 cm, 10 cm, 15 cm, 20 cm, 25 cm, 30 cm, and an unmowed control treatment. Mowing treatments were applied three times throughout the study duration, followed by a final harvest. Data were collected on aboveground plant productivity, plant community diversity, and levels of soil carbon, nitrogen, and organic matter. Results showed plant productivity to be greatest at lower cutting heights, decreasing as cutting height increased. M0, M5, and M10 treatments produced over 300% more cumulative biomass than the control treatment. There were no differences across mowing treatments for measures of species diversity. The ten-centimetre treatment produced highest values of soil carbon, nitrogen, and organic matter than many other mowing treatments after three treatment applications (p <  0.05). Results indicate that lower cutting heights produced higher levels of aboveground biomass, did not alter crop species composition throughout the course of the study, and have potential to contribute towards the carbon pool. These results provide insight on the use of mowing within perennial cropping systems, and the effects on aboveground productivity and levels of soil carbon. The implications of this study allow agricultural producers to make informed decisions on how to manage their land for optimum productivity and environmental sustainability.

## 1. Introduction

Agricultural producers typically utilize mechanical mowing as a method of harvesting their crops. This technique has important management implications for agricultural land. Through defoliation, mowing can stimulate compensatory growth within plants, facilitating the production of new roots and shoots [1,2]. Compensatory growth is defined as a growth response that allows for a plant to counteract the tissue lost to defoliation through the production of new plant material [1]. Defoliation represents a massive decrease in a plant’s ability to photosynthesize as a large amount of leaf area is removed. Compensatory growth can return the plant’s function to pre-defoliation conditions, thus nutrient allocation is shifted to produce new plant material to increase photosynthetic capacity [1,3].

Mowing practices also have potential to impact plant species diversity, depending on mowing intensity. Through defoliation of dominant species, mowing can reduce levels of competition, resulting in growth of less competitive plant species and increases in biodiversity [4,5], with maximum diversity occurring at levels of moderate defoliation [6]. Low mowing heights can cause increased levels of defoliations that may damage plants and lower species diversity [7]. Using a higher cutting height does not reduce competition compared to lower cutting heights, and thus does not favour increases in biodiversity [8]. Ultimately, species diversity appears to vary as a function of mowing intensity, suggesting this practice can be used to maintain or improve species diversity, depending on the management goals of a cropping system.

In addition to affecting plant productivity and community dynamics, mowing can also result in changes in soil chemistry [9]. Recent studies have shown that mowing can increase levels of soil carbon and nitrogen compared to areas left unmowed [10]. Regular mowing encourages plant productivity and root exudates, which in turn contribute towards soil stocks of carbon and nitrogen [11]. These soil variables differ depending on mowing duration and frequency [11] and so different mowing practices have potential to differentially alter soil chemistry. Soils provide integral ecosystem services, contributing towards carbon storage and providing humanity with nearly 99% of its food [12], making it one of the world’s most valued resources. It is therefore important to understand how different management practices can affect soil chemistry. Understanding how different mowing intensities affect plant productivity and soil properties will allow producers to manage agricultural land for optimum productivity and sustainability.

Using mowing advantageously to increase production of forage crops not only provides higher crop yields, but also gives producers the opportunity to combat their greenhouse gas (GHG) emissions by the potential to sequester atmospheric carbon within the soil and in plant material [13,14]. The amount of soil organic matter and vegetation present on agricultural land is positively correlated with the amount of carbon sequestration occurring [15]. If producers implement mowing strategies to increase production, they are also increasing the amount of carbon dioxide being captured from the atmosphere by their crops, helping to potentially turn their agricultural land into a carbon sink [13,16].

This paper explores the effect of mowing height on the plant and soil characteristics of a perennial cropping system in the central interior of British Columbia, Canada. This experiment investigates how varying mowing heights affect 1) aboveground plant productivity, 2) plant community diversity, and 3) levels of soil carbon and nutrients of a perennial cropping system, helping to address the knowledge gap of how mowing height impacts these soil parameters and provide information on how this agricultural practice may become more sustainable. It was hypothesized that i) primary production would be highest in areas that are subject to an intermediate mowing height, as highest levels of compensatory growth are achieved with intermediate defoliation, as seen in previous studies [7,17,18], ii) species diversity would be greatest at intermediate mowing heights as previous studies have shown this mowing intensity favours species diversity [6], and iii) higher levels of carbon sequestration would correspond with the most productive mowing treatments, as productivity is positively correlated with carbon sequestration [13,15,16].

## 2. Methods

### 2.1 Study site and experimental design

**Fig 1:**
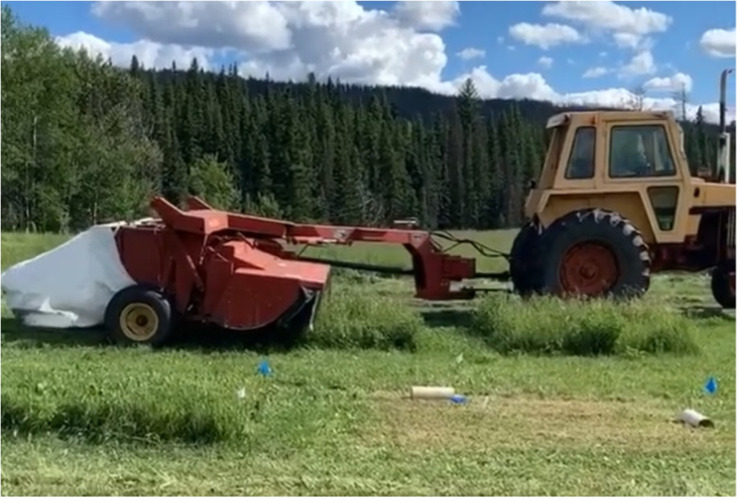
Tractor and mower, fit with collection bag, implementing the mowing treatments in the study enclosure in June 2021.

This study was conducted in the Central Interior region of British Columbia, Canada. The study area receives approximately 493 mm of annual precipitation, has an average annual temperature of 4.0° C [19], and an elevation of 777 m. The research site was on an agricultural field used for hay production (52.08252º N, -123.52085º W). In 2009, the field was tilled and seeded with a perennial forage blend of *Phleum pratense*, *Medicago sativa*, and *Trifolium pratense*, with an application rate of 21.3 kg/ha. Irrigation was delivered to the study area by a centre pivot sprinkler system which typically runs annually from May to September, all study plots were irrigated similarly throughout the study’s duration. Irrigation was applied as needed throughout the season. The irrigated cropland was fertilized annually in May with an inorganic fertilizer composed of 50% nitrogen, 20% phosphorus, 20% potash, and 10% sulphur, at an application rate of 214.6 kg/ha. In April 2020, a study enclosure was established on this field, measuring approximately 10 m x 100 m, divided into six blocks, representing six replicates. Each block was organized into 2 m x 2 m plots, including 1 m buffer strips between plots. Each plot was randomly assigned one of eight experimental treatments. The treatments included stubble heights of 0 cm (M0), 5 cm (M5), 10 cm (M10), 15 cm (M15), 20 cm (M20), 25 cm (M25), 30 cm (M30), and a no-mowing control treatment (C). Biweekly mowing of the buffer strips between plots was carried out using a Husqvarna 160cc 3-in-1 Push Lawn Mower to ensure plot perimeters were kept visible. Experimental mowing treatments were applied in June 2020, August 2020, and June 2021. In August 2021, a final harvest occurred, harvesting all plots to a stubble height of 0 cm. Mowing treatments were achieved using a New Holland 411 discbine mower pulled behind 1974 Case 870 tractor. A large bag was attached to the deflector panels of the mower to capture all cut material (Fig 1). For each mowing treatment, the deck of the mower was lowered to the appropriate height by the hydraulics of the tractor, using a ruler to then manually measure the cutting height. The mowing height was first tested on portions of the field outside of the experimental plots to ensure proper treatment heights were achieved. The height of the mower deck was measured and readjusted, if needed, between mowing of each individual plot. M0 plots were cut at a 5 cm cutting height and then mowed on the lowest setting of a Husqvarna 160cc 3-in-1 Push Lawn Mower to ensure the vegetation was taken down to the lowest height possible (approximately 2 cm). Cut biomass was emptied from the bag in an area outside of the study enclosure. Any cut biomass that had not been collected was manually raked and removed from the study enclosure. After mowing, vegetation height within each plot was measured to ensure it had been cut at the proper height. If there were any discrepancies, adjustments were made manually using garden shears. Control treatment plots were not mowed throughout the duration of the study.

### 2.2 Field sampling and measurements

Vegetation sampling was completed before each treatment application from two 0.5 m x 0.5 m quadrat frames randomly placed within each plot. Aboveground biomass was collected from each frame at each plot’s corresponding treatment height. Biomass samples were collected immediately before mowing treatments were applied, using a ruler to measure to treatment height and gardening shears to cut plant material. Values of species richness and Shannon-Weiner diversity [20] were determined for each 0.5 m x 0.5 m frame prior to biomass collection. After these samples and data were collected, mowing treatments were applied to study plots. At the final harvest, additional biomass sampling occurred to harvest the remaining vegetation within each plot to a height of 0 cm. Once collected, biomass samples were placed in paper bags and dried for 48 hours at 65 °C after which they were weighed to determine total dry sample weight. Cumulative biomass for each mowing treatment was determined by the summation of biomass values from all four sampling events and final harvest.

Baseline and final soil samples were collected at a depth of 0 - 15 cm. A spade shovel was used to dig a hole approximately 15 cm in both diameter and depth. Soil was then collected by scraping the sides of the hole. Baseline soil sampling occurred in May 2020, prior to any treatment applications. Two 10 m transects were sampled at 2 m increments in each block, for a total of 10 soil samples per block. Following the final harvest in August 2021, final soil samples were taken from within the vegetation sampling frames at a depth of 0 - 15 cm. Soils from the two sampling locations were combined, resulting in one sample for each study plot and six samples for each mowing treatment. As baseline and final soil samples were collected from different locations and at different times of the year, soil samples were only compared within sampling events.

Fresh soil was used to determine levels of soil organic matter (SOM). Soil was first dried for sixteen hours at 105 °C using a Yamato™ DKN812 drying oven then weighed to determine dry soil weight. Samples were then burned at 500 °C for five hours in a Barnstead Thermolyne 62700 furnace after which they were weighed for a second time. Levels of SOM were reported as a percentage of total soil volume (% SOM), calculated using Equation 1 [21]. To determine total soil carbon and nitrogen, subsamples of soil samples were air-dried until they were a constant weight and then passed through a 2 mm sieve. Three replicates of each dried, sieved sample, weighing between 10 - 15 mg, were folded into aluminum capsules. Prepared samples were then analyzed for levels of total carbon and total nitrogen using a ThermoScientific™ FlashSmart Elemental Analyzer [21,22]. In addition to examining each technical replicate against one another, Organic Analytical Standards (OAS) with known nitrogen and carbon values provided by ThermoScientific™ were analyzed to ensure accurate estimation of elemental analysis. Levels of soil carbon and nitrogen are reported as total carbon, total nitrogen, and the carbon-to-nitrogen (C:N) ratio.


$$\%SOM=\left[\frac{\left(105OvenWeight-500MufflerWeight\right)}{105OvenWeight}\right]x100$$ (1)

### 2.3 Data analysis and statistics

All statistical analyses and figures were produced using R for Statistical Computing, version 4.0.3 [23]. Plant productivity, species diversity, and soil characteristic responses were compared between mowing treatments using blocked ANOVAs. All models included study block as a random value. Tukey’s HSD post hoc analyses were performed on all models using the “emmeans” package. In all cases, significance was defined by p <  0.05. All models were examined to ensure they met the assumptions of normally distributed residuals and homogeneity of variance. If these assumptions were not met, data transformations were performed. 2021 total carbon and SOM data were transformed using a log transformation. 2021 total nitrogen data were transformed using a log +  3 transformation.

## 3. Results

### 3.1 Aboveground plant productivity

**Fig 2:**
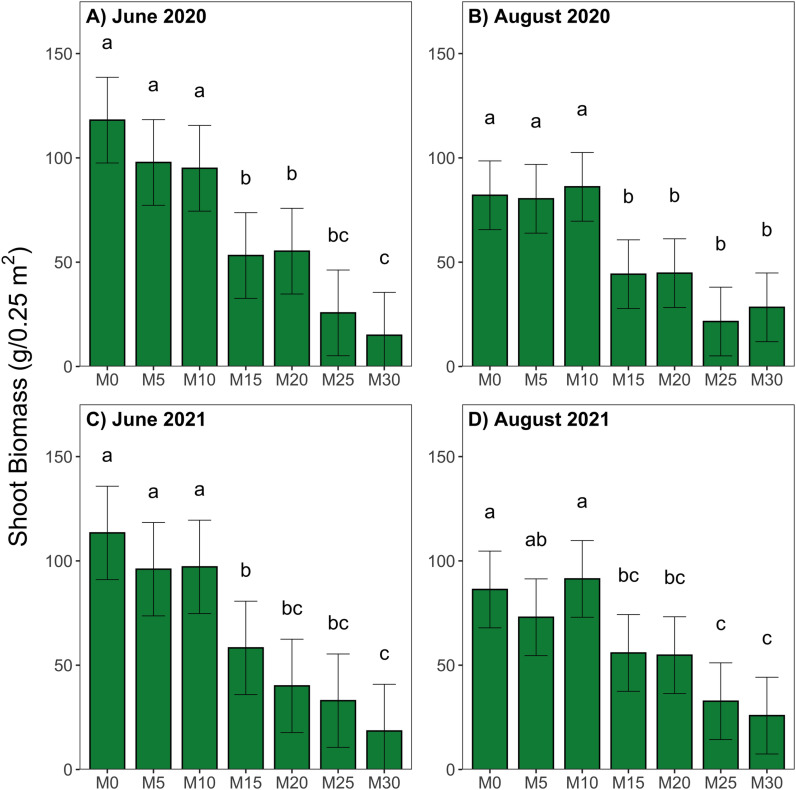
Aboveground biomass samples collected across mowing treatments in A) June 2020; B) August 2020; C) June 2021; and D) August 2021, comparisons made within sampling events, error bars represent 95% confidence intervals, significant differences denoted by different lowercase letters as determined by post-hoc Tukey test following ANOVA (p < 0.05, n = 12).

Mowing treatments showed significant differences in aboveground biomass values at all sampling events (p <  0.05, Fig 2), with aboveground biomass decreasing as cutting height increased. Highest levels of aboveground biomass were seen at the lower cutting treatments of M0, M5 and M10. In August of both sampling years, average aboveground biomass collected from M10 treatment plots, though not significant, was slightly higher than the lower cutting treatments M0 and M5. Mowing treatments can generally be grouped by similar yield into low (M0, M5 and M10), moderate (M15 and M20), and high (M25 and M30) cutting height categories.

**Fig 3:**
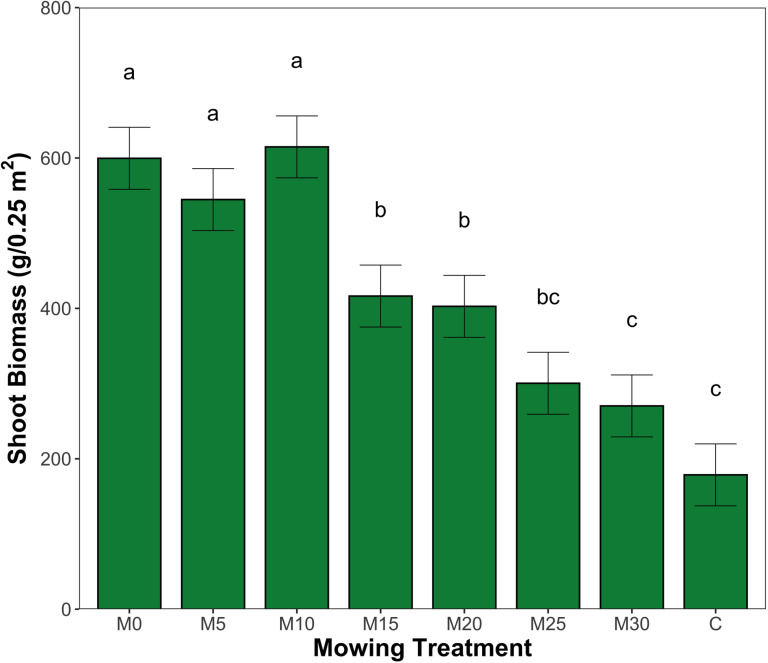
Cumulative aboveground biomass harvested across mowing treatments from June 2020 to August 2021, including final harvest to 0 cm, error bars represent standard error, significant differences are denoted by different lowercase letters as determined by post-hoc Tukey test following ANOVA (p <  0.05).

Cumulative aboveground biomass across mowing treatments throughout the study duration showed similar results to individual sampling events. Low mowing heights had significantly higher levels of cumulative aboveground biomass while the higher cutting heights and control treatment expressed lower values of productivity (p <  0.05, Fig 3). M10 produced highest levels of cumulative aboveground biomass, the control treatment showed the lowest.

### 3.2 Plant species diversity

**Fig 4:**
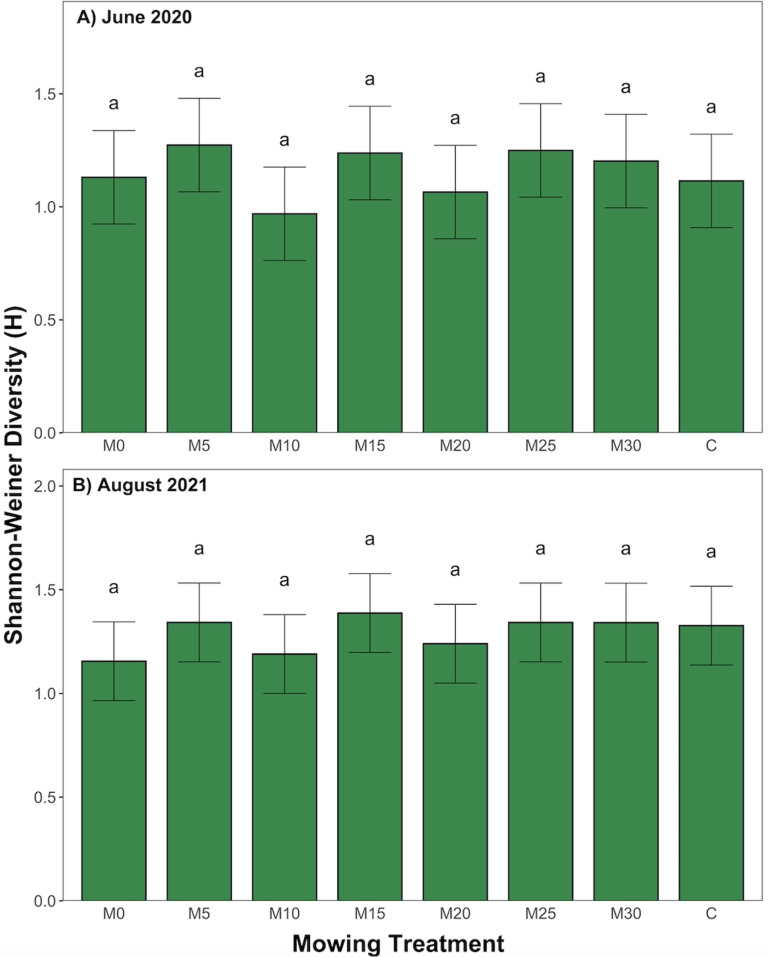
Comparison of Shannon-Weiner Diversity across mowing treatments in A) June 2020 and B) August 2021, error bars represent standard error, significant differences are denoted by different lowercase letters as determined by post-hoc Tukey test following ANOVA (p <  0.05).

No significant differences were found in Shannon-Weiner Diversity across experimental plots prior to treatment application in June 2020 or in August 2021, after three treatment applications (Fig 4A and 4B, p <  0.05). June 2020 H’ values ranged from 0.000 to 1.618, with a mean of 1.155. H’ values in August 2021 ranged from 0.000 to 1.816, with a mean value of 1.291.

### 3.3 Soil carbon, nitrogen, and organic matter

**Fig 5:**
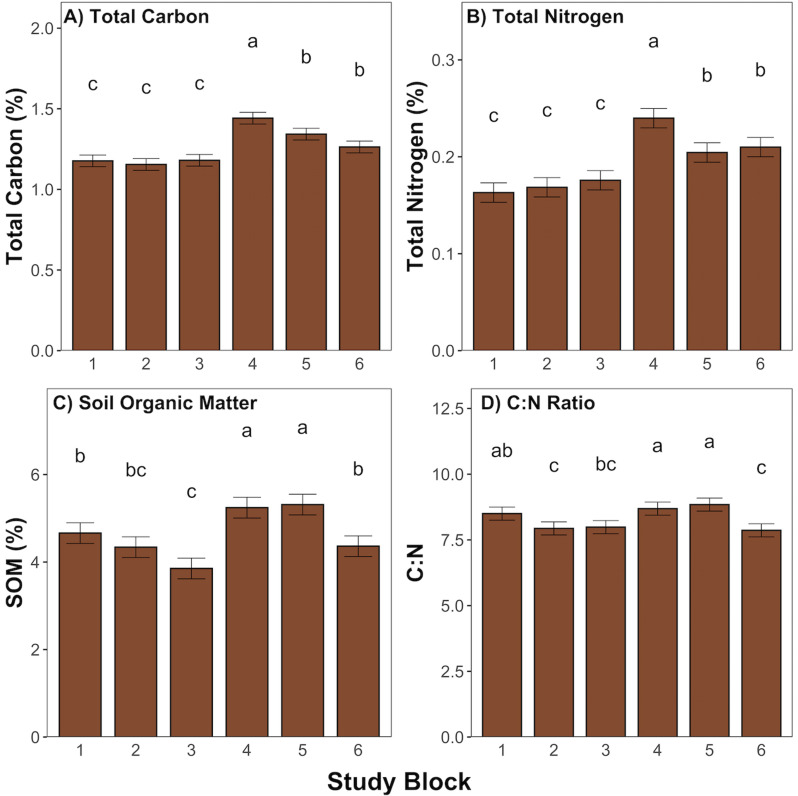
Comparison of soil metrics across study blocks collected in May 2020 at a depth of 0 to 15 cm; A) Total Carbon; B) Total Nitrogen; C) Soil Organic Matter; D) C: N Ratio, error bars represent standard error, significant differences are denoted by different lowercase letters as determined by post-hoc Tukey test following ANOVA (p <  0.05).

Soils collected in 2020 and 2021 were analyzed for levels of total carbon, total nitrogen, SOM, and the C:N ratio. Fig 5 displays the comparison between study blocks at the time of baseline soil sampling (May 2020) for these soil variables. There is inherent variation throughout the study blocks, with blocks 1, 2, and 3 tending to have significantly lower values (p <  0.05) for total carbon (Fig 5A, p <  0.05) and nitrogen (Fig 5B, p <  0.05) than other blocks. Blocks 4 and 5 showed significantly higher values of SOM (Fig 5C, p <  0.05) and the C:N ratio (Fig 5D, p <  0.05) than other blocks

**Fig 6:**
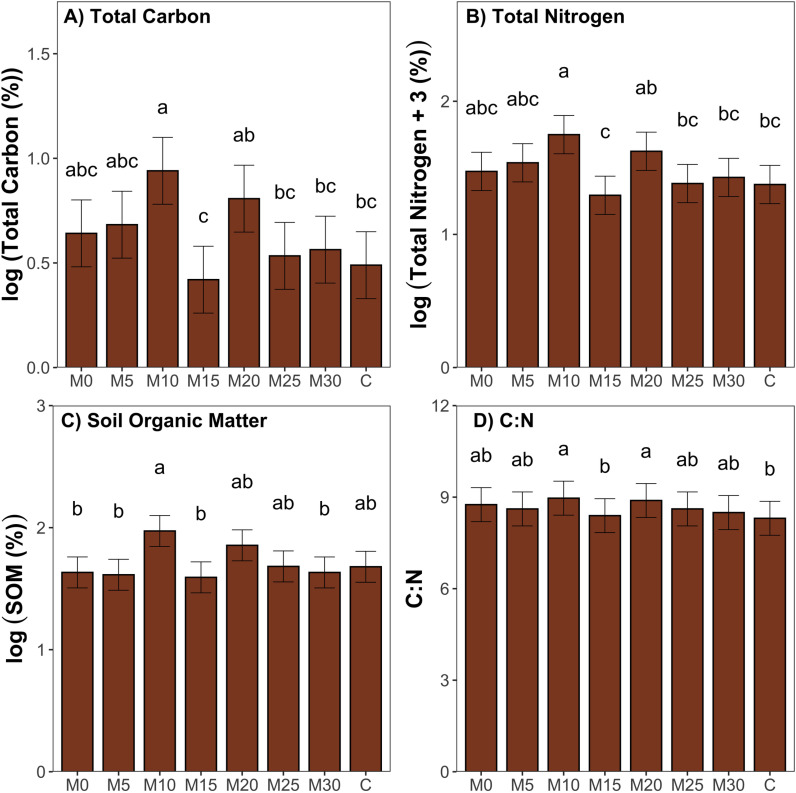
Comparison of soil metrics across mowing treatments collected in August 2021 at a depth of 0 to 15 cm; A) Total Carbon; and B) Total Nitrogen, C) Soil Organic Matter, and D) C:N Ratio, error bars represent standard error, significant differences are denoted by different lowercase letters as determined by post-hoc Tukey test following ANOVA (p <  0.05).

Fig 6 shows a comparison between mowing treatments for soil variables at final soil sampling performed in August 2021. M10 was shown to have greater values of both total carbon (Fig 6A, p <  0.05) and total nitrogen (Fig 6B, p <  0.05) than M15, M25, M30, and control treatments. Fig 6A and 6B also show that M15 had significantly lower values of total carbon and total nitrogen than the M10 and M20 treatments (p <  0.05). SOM, shown in Fig 6C, was found to be significantly higher at M10 than at M0, M5, M15, and M30 (p <  0.05). The M10 treatment also saw significantly higher values for the C:N ratio when compared to M15 and control treatments (Fig 6D, p <  0.05). For all soil variables shown in Fig 6 (total carbon, total nitrogen, SOM, and the C:N ratio), highest values are shown for the M10 treatment.

## 4. Discussion

### 4.1 Aboveground plant productivity

Mowing is shown to produce a compensatory growth response and stimulate plant productivity, both in this study and in many previous [8,24–27]. The compensatory growth response is thought to be due to high levels of cytokinins, which are plant hormones within root systems that promote cell division [1,28]. Defoliation increases the root to shoot ratio, and thus alters concentrations of cytokinins within a plant. The cytokinins remaining in the root system are transported to remaining aboveground plant tissue where they stimulate cell division and allow for plant regrowth following defoliation [29]. Production of a compensatory growth response is dependent upon many factors, including the amount of photosynthetic material remaining after defoliation, levels of carbohydrate reserves within rooting systems, phenological stage at time of defoliation, and resource availability following defoliation [1,3]. Because of the dependence of compensatory growth on all these factors, levels of this mechanism vary with defoliation intensity and timing, species composition, and environmental conditions.

A plant’s regrowth response to mowing, or degree of compensatory growth, is important for the continued plant production and yield of a cropping system [24]. Previous studies have investigated mowing height as a determining factor in how plants respond to this practice. Decreased levels of compensatory growth were seen when using mowing heights which left a stubble height lower than 6 cm [17] and removed 80% of aboveground plant parts [18]. These low cutting heights remove large proportions of aboveground biomass, which can increase bare ground exposure and reduce the amount of nutrient return to the soil [17,25], resulting in unfavourable conditions for both plant and soil communities. Low mowing heights may produce high levels of defoliation that some plants may not be able to recover from [7]. These studies have found that high cutting heights caused less damage to plants, leaving adequate resources for them to produce higher levels of compensatory growth [18]. However, leaving a high stubble height may eventually lead to decreased long-term regrowth through build-up of plant litter, and thus decreased biomass production [8]. Mowing heights that are either too low or too high can both have negative impacts on the plant productivity of agricultural land.

In this study, aboveground plant productivity, estimated by shoot biomass, was shown to be highest at low mowing heights, with productivity trending downwards as mowing height increased. M0, M5, and M10 appeared favourable for a high yielding first harvest in June, stimulating the maximal compensatory growth response for a second high yielding harvest in August. Higher mowing treatments (M15 through M30) produced lower biomass volumes at all sampling events. Lowest aboveground productivity, throughout both study years, was seen in control plots. These unmowed plots showed the lowest levels of cumulative biomass compared to any of the mowing treatments. This disparity in productivity is likely due to the absence of a compensatory growth response as these plots did not experience any defoliation throughout the study’s duration. As biomass accumulated in these plots, the amount of shading and light competition between plants increased, this could also attribute to the reduction of productivity seen for this treatment [30]. This shows that while mowing is often a necessary practice for agricultural operations, it can also be beneficial to help increase plant productivity through the mechanism of compensatory growth.

The differences in aboveground productivity seen between mowing treatments are contrary to the hypothesis that intermediate heights would be most productive and contradict the findings of many previous studies which closely examined cutting heights [2,8,17,18,24,31]. Each of these studies identified a moderate mowing height, ranging from 14 cm to 24 cm, to produce highest levels of aboveground productivity. These previous studies have been performed in both natural and controlled, irrigated environments but examined much different plant species. This study’s results opposing others’ is likely due to crop specificity and current management regimes, as these both affect how a cropping system will respond to mowing [2].

All plants greatly vary in how they respond to defoliation [2]. The main components of this study’s cropping system were *P. pratense*, *T. pratense*, and *M. sativa*. While *P. pratense* regrows best when cut at heights above 10 cm [32], both *T. pratense* and *M. sativa* are able to respond to lower cutting heights, yielding a higher regrowth response when a cutting height less than 5 cm is used. The ability to respond to heavy defoliation and large reductions in photosynthetic capacity is attributed to carbohydrate reserves within legume tap roots, which are allocated towards the new production of photosynthetic material [33–35]. These two legume species make up the majority of the plant community throughout the study area, resulting in a cropping system that responds best to lower cutting heights, specifically those under 10 cm.

Irrigation and fertilization of the study site are likely also contributing to the observed response to mowing treatments. *P. pratense, T. pratense, and M. sativa* respond well to irrigation, producing higher yields under moist conditions [32,34,36,37]. The fertilization combination of nitrogen, phosphorus, and potash annually applied to this cropping system facilitates high yields at both first and second harvest, providing essential nutrients required for successful growth of legume species [34,36,38]. Phosphorus and potash promote root development which is important for carbohydrate storage and regrowth responses in *T. pratense* and *M. sativa* [36]. Nitrogen aids the growth of *P. pratense* [39], allowing it to compete with the legume species during the early growing season [32]. The irrigation and fertilization management in place at the study site provides the necessary water and nutrients required for each species to be highly productive and produce a strong compensatory growth response to mowing. In areas where moisture or nutrients are more limited, a different response to mowing may be experienced.

### 4.2 Plant species diversity

Mowing has been shown to decrease or increase plant diversity depending on intensity [8,17, 40], however this was not found in the results presented here. Contrary to our hypothesis and the results of previous studies, mowing treatments did not change levels of plant diversity throughout the study’s duration. Current management goals and previous management history of the study area are likely responsible for the consistent levels of diversity seen throughout multiple treatment applications.

Management goals of perennial cropping systems tend to focus on productivity rather than species diversity and thus are typically seeded with either monocultures or simple grass-legume mixtures [41]. Species are selected for their relative yield, nutrient content, and adaptability to environmental conditions [42]. The success of a forage system is determined by the high cover and yield of seeded forage species, rather than levels of biodiversity. Invasion of unseeded, unpalatable species, which may increase diversity, are unwanted in these systems and can represent a reduction in either crop or nutrient yield [43]. The species composition of the study site reflects this goal, with a high cover of seeded forage species, and relatively low cover and abundance of other species.

Management practices applied to established perennial cropping systems can further promote the success of desired forage species. Irrigation and fertilization have been consistently used at the study site and are attributing to the success of seeded species. These practices help to decrease water and nutrient restrictions, ultimately increasing biomass production. The combination of these techniques has been shown to decrease levels of species diversity [44]. In this cropping system, diversity is maintained through the success and abundance of seeded forage species. The results of this study are only applicable when considering the same, or similar, forage species. Future studies should continue to explore the effects of mowing height on both aboveground productivity and species diversity of other forage crops, to gain a more comprehensive understanding of mowing as an agricultural practice.

### 4.3 Soil carbon, nitrogen, and organic matter

Changes in plant productivity typically correlate with changes in soil carbon, as aboveground productivity is a primary source of soil carbon [45]. Therefore, management techniques that affect productivity can also be used to influence levels of soil carbon [15]. Mowing has been shown to effect plant productivity, however most short-term studies do not account for the direct correlation between mowing and soil variables, as soil takes longer to respond to management changes than the plant community [17]. This study attempted to evaluate the soil response to mowing by comparing soil variables across mowing treatments. Inherent variation was seen throughout study blocks when considering baseline soil analyses. Even prior to treatment application, there were significant differences seen in soil variables from one block to another. Soil properties can vary across agricultural fields according to topography, soil texture, and management practices [46,47]. This effect, combined with the short-term nature of this study, did not allow for strong conclusions to be drawn when considering the soil response to mowing treatments. Interestingly, when analyzing the post-treatment soil samples, the M10 treatment saw higher levels of total carbon, total nitrogen, soil organic matter and the C:N ratio compared to various other treatments, which supports the hypothesis that higher levels of carbon sequestration would correspond with the most productive mowing treatments. These increases could be a result of the improvements in aboveground productivity also seen for the M10 treatment. However, M0 and M5 treatments, which also showed increased aboveground productivity, did not produce corresponding increases in soil nutrients. While previous studies have shown a correlation between aboveground plant productivity and soil carbon [13,45,48], there is not enough evidence provided in this study to reinforce this finding and fully support our hypothesis. The changes in soil variables seen for the M10 treatment, after only two seasons of treatment application, suggest potential for mowing to influence levels of carbon sequestration, however longer term studies should be performed to confirm the trend seen in this study and to fully evaluate mowing as a technique applied to perennial cropping systems.

## 5. Conclusion

This study has shown that perennial cropping systems majorly composed of agronomic legume species, when provided with sufficient resources, such as irrigation and fertilization, can withstand repeated heavy defoliation, continuing to produce high yields under these conditions, without experiencing changes in species diversity. Utilizing a mowing height less than or equal to 10 cm has been shown to be favourable for increased levels of aboveground productivity. Over this two-year study, the 10 cm mowing treatment produced highest levels of cumulative aboveground biomass across all treatments and showed potential for reciprocal increases in soil carbon and nitrogen. These findings suggest that mowing is not only a method to harvest forage crops but can also be used for mutual benefits to producers and the environment through increased production and carbon sequestration. This study suggests that with proper mowing management, the forage production industry can improve its sustainability and productivity simultaneously. The information in this study can be used by producers across the globe to make informed decisions on how to manage their cropping systems for these environmental and yield benefits. Future research should examine the effects of continued application of mowing treatments to ensure the results seen in this study continue over time. Additional research should be performed on different forage cropping systems throughout various climates across the world, to gain a comprehensive understanding of mowing as an agricultural practice. Longer term studies would provide more insight as to how soil properties respond to mowing.

## Supporting information

S1 File S1_Dataset_Bayliff et al. (XLSX)

## References

[1] McNaughton SJ. Compensatory Plant Growth as a Response to Herbivory. Oikos. 1983;40(3):329. doi: 10.2307/3544305

[2] Huhta A-P, Hellström K, Rautio P, Tuomi J. Grazing tolerance of Gentianella amarella and other monocarpic herbs: why is tolerance highest at low damage levels?. Plant Ecology. 2003;166(1):49–61. doi: 10.1023/a:1023278502972

[3] Briske DD, Richards JH. 1995. Plant responses to defoliation: A physiological, morphological and demographic evaluation Wildland Plants: Physiological Ecology and Developmental Morphology. Wildland Plants: physiological ecology and developmental morphology. 635–710.

[4] Grime JP. Evidence for the Existence of Three Primary Strategies in Plants and Its Relevance to Ecological and Evolutionary Theory. The American Naturalist. 1977;111(982):1169–94. doi: 10.1086/283244

[5] Koerner SE, Smith MD, Burkepile DE, Hanan NP, Avolio ML, Collins SL, et al. Change in dominance determines herbivore effects on plant biodiversity. Nat Ecol Evol. 2018;2(12):1925–32. doi: 10.1038/s41559-018-0696-y 30374174

[6] Grime JP. 1973. Control of species density in herbaceous vegetation. Journal of Environmental Management 1: 151–167.

[7] Tälle M, Bergman K-O, Paltto H, Pihlgren A, Svensson R, Westerberg L, et al. Mowing for biodiversity: grass trimmer and knife mower perform equally well. Biodivers Conserv. 2014;23(12):3073–89. doi: 10.1007/s10531-014-0765-8

[8] Wan Z, Yang J, Gu R, Liang Y, Yan Y, Gao Q, et al. Influence of Different Mowing Systems on Community Characteristics and the Compensatory Growth of Important Species of the Stipa grandis Steppe in Inner Mongolia. Sustainability. 2016;8(11):1121. doi: 10.3390/su8111121

[9] Gillespie MAK, Buckley HL, Condron L, Wratten SD. Grassland plant and invertebrate species richness increases from mowing are mediated by impacts on soil chemistry. Basic and Applied Ecology. 2022;63 152–63. doi: 10.1016/j.baae.2022.06.010

[10] Hyvönen T, Huusela E, Kuussaari M, Niemi M, Uusitalo R, Nuutinen V. Aboveground and belowground biodiversity responses to seed mixtures and mowing in a long-term set-aside experiment. Agriculture, Ecosystems & Environment. 2021;322:107656. doi: 10.1016/j.agee.2021.107656

[11] Li Y. Beam deflection and scanning by two-mirror and two-axis systems of different architectures: a unified approach. Appl Opt. 2008;47(32):5976–85. doi: 10.1364/ao.47.005976 19002221

[12] Kopittke PM, Menzies NW, Wang P, McKenna BA, Lombi E. Soil and the intensification of agriculture for global food security. Environ Int. 2019;132:105078. doi: 10.1016/j.envint.2019.105078 31400601

[13] Conant RT, Paustian K, Elliott ET. Grassland management and conversion into grassland: effects on soil carbon. Ecological Applications. 2001;11(2):343–55. doi: 10.1890/1051-0761(2001)0110343:gmacig.2.0.co;2

[14] Bai Y, Cotrufo MF. Grassland soil carbon sequestration: Current understanding, challenges, and solutions. Science. 2022;377(6606):603–8. doi: 10.1126/science.abo2380 35926033

[15] Mattila TJ, Vihanto N. Agricultural limitations to soil carbon sequestration: Plant growth, microbial activity, and carbon stabilization. Agriculture, Ecosystems & Environment. 2024;367:108986. doi: 10.1016/j.agee.2024.108986

[16] Jansson C, Faiola C, Wingler A, Zhu X-G, Kravchenko A, de Graaff M-A, et al. Crops for Carbon Farming. Front Plant Sci. 2021;12:636709. doi: 10.3389/fpls.2021.636709 34149744 PMC8211891

[17] Yang Z, Minggagud H, Baoyin T, Li FY. Plant production decreases whereas nutrients concentration increases in response to the decrease of mowing stubble height. J Environ Manage. 2020;253:109745. doi: 10.1016/j.jenvman.2019.109745 31671323

[18] Zhao W, Chen S-P, Lin G-H. Compensatory growth responses to clipping defoliation in Leymus chinensis (Poaceae) under nutrient addition and water deficiency conditions. Plant Ecol. 2007;196(1):85–99. doi: 10.1007/s11258-007-9336-3

[19] Moore RD, Spittlehouse RL, Whitfield PH, Stahl K. 2010. Weather and climate. Government of British Columbia. https://www.for.gov.bc.ca/hfd/pubs/Docs/Lmh/Lmh66/Lmh66_ch03.pdf

[20] Spellerberg IF, Fedor PJ. A tribute to Claude Shannon (1916–2001) and a plea for more rigorous use of species richness, species diversity and the ‘Shannon–Wiener’ Index. Global Ecology and Biogeography. 2003;12(3):177–9. doi: 10.1046/j.1466-822x.2003.00015.x

[21] Gavlak RG, DA Horneck, O Miller. 2005. Soil, plant and water reference methods for the Western region (3rd ed.). WREP-125, WERA-103 Technical Committee. 207pp.

[22] ThermoScientific. 2017. FlashSmart elemental analyzers. Thermo Fisher Scientific. 94pp.

[23] R Core Team. 2021. R: A language and environment for statistical computing. R Foundation for Statistical Computing. Vienna, Austria. URL https://www.R-project.org/.”

[24] Wang X, Guo X, Hou X, Zhao W, Xu G, Li Z. Effects of leaf zeatin and zeatin riboside induced by different clipping heights on the regrowth capacity of ryegrass. Ecological Research. 2013;29(2):167–80. doi: 10.1007/s11284-013-1107-0

[25] Han X, Sistla SA, Zhang Y-H, Lü X-T, Han X-G. Hierarchical responses of plant stoichiometry to nitrogen deposition and mowing in a temperate steppe. Plant Soil. 2014;382(1–2):175–87. doi: 10.1007/s11104-014-2154-1

[26] Luo X, Li J, Xie Y, Wang Y, Yu J, Liang X. Enhanced Primary Productivity in Fenced Desert Grasslands of China through Mowing and Vegetation Cover Interaction. Agronomy. 2023;13(8):2029. doi: 10.3390/agronomy13082029

[27] Ma Y, Zheng Q, Zhang Y, Ganjurjav H, Yue H, Wang X, et al. Short-term robust plant overcompensatory growth was observed in a degraded alpine meadow on the southeastern Qinghai-Tibetan Plateau. Sci Total Environ. 2024;918:170607. doi: 10.1016/j.scitotenv.2024.170607 38336057

[28] Jiang P, Han P, He M, Shui G, Guo C, Shah S, et al. Appropriate mowing can promote the growth of Anabasis aphylla through the auxin metabolism pathway. BMC Plant Biol. 2024;24(1):482. doi: 10.1186/s12870-024-05204-3 38822275 PMC11141038

[29] Shah S, Cai L, Li X, Fahad S, Wang D. Influence of cultivation practices on the metabolism of cytokinin and its correlation in rice production. Food and Energy Security. 2023;12(5). doi: 10.1002/fes3.488

[30] Martinez-Garcia JF, Rodriguez-Concepcion M. Molecular mechanisms of shade tolerance in plants. New Phytol. 2023;239(4):1190–202. doi: 10.1111/nph.19047 37282777

[31] Turner CL, Seastedt TR, Dyer MI. 1993. Maximization of aboveground grassland production: the role of defoliation frequency, intensity, and history. Ecological Applications. 3(1):175–186. http://doi.wiley.com/10.2307/1941800 10.2307/1941800 27759215

[32] Ogle DG, St. John L and DJ Tilley. 2011. Plant guide for timothy (*Phleum pratense*). USDA-Natural Resources Conservation Service, Idaho State Office. Boise, ID. https://plants.usda.gov/DocumentLibrary/plantguide/pdf/pg_phpr3.pdf

[33] Wiersma D, Bertam M, Wiederholt R, Schneider N. 2007. The long and short of alfalfa cutting height. Focus on Forage. Focus on Forage. https://fyi.extension.wisc.edu/forage/files/2014/01/AlfCutHt.pdf

[34] St. John L and Ogle D. 2008. Plant guide – Red clover (*Trifolium pratense* L.). United States Department of Agriculture. https://plants.sc.egov.usda.gov/DocumentLibrary/plantguide/pdf/pg_trpr2.pdf

[35] Bai J, Zhang Y, Liu X, Feng W, Li Q, Long M, et al. Integrated transcriptomic and metabolomic analyses reveals the molecular bases of alfalfa regrowth processes of new shoots after cutting under different water and nitrogen availability. Industrial Crops and Products. 2024;213:118476. doi: 10.1016/j.indcrop.2024.118476

[36] Undersander D, Cosgrove D, Cullen E, Grau C, Rice ME, Renz M, Sheaffer C, Shewmaker G, Sulc M. 2011. Alfalfa management. University of Wisconsin Extension. 68.

[37] Kamran M, Yan Z, Jia Q, Chang S, Ahmad I, Ghani MU, et al. Irrigation and nitrogen fertilization influence on alfalfa yield, nutritive value, and resource use efficiency in an arid environment. Field Crops Research. 2022;284:108587. doi: 10.1016/j.fcr.2022.108587

[38] Baidoo M. Alfalfa response to phosphorus and potassium fertility in relation to calcium and magnesium. International Journal on Agriculture Research and Environmental Sciences. 2024;5(1):1–7. doi: 10.51626/ijares.2024.05.00040

[39] Wiśniewska-Kadżajan B, Malinowska E. Changes in the Nutritional Value of Timothy (Phleum pratense L.) Biomass as a Response to Varied Organomineral Treatment. 2024. doi: 10.21203/rs.3.rs-3937870/v1

[40] Li G, Barfknecht DF, Gibson DJ. Disturbance effects on productivity–plant diversity relationships from a 22‐year‐old successional field. J Vegetation Science. 2020;32(1). doi: 10.1111/jvs.12970

[41] Sanderson MA, Goslee S, Soder K. 2013. Biodiversity in forage stands. Farmwest. https://farmwest.com/wp-content/uploads/2020/08/9-AFM-2013-09-Sanderson.pdf

[42] Milić D, Katanski S, Milošević B, Živanov D. Variety selection in intensive alfalfa cutting management. Ratar i povrt. 2019;56(1):20–5. doi: 10.5937/ratpov56-20528

[43] Bittman S, McCartney D, Hunt D, Waddington J. The value of pasture weeds. Farmwest. 2013.

[44] Müller IB, Buhk C, Lange D, Entling MH, Schirmel J. Contrasting effects of irrigation and fertilization on plant diversity in hay meadows. Basic and Applied Ecology. 2016;17(7):576–85. doi: 10.1016/j.baae.2016.04.008

[45] Kunkel ML, Flores AN, Smith TJ, McNamara JP, Benner SG. A simplified approach for estimating soil carbon and nitrogen stocks in semi-arid complex terrain. Geoderma. 2011;165(1):1–11. doi: 10.1016/j.geoderma.2011.06.011

[46] Usowicz B, Lipiec J. Spatial variability of soil properties and cereal yield in a cultivated field on sandy soil. Soil and Tillage Research. 2017;174:241–50. doi: 10.1016/j.still.2017.07.015

[47] Richardson GS, Ruark MD, Radatz T, Radatz A, Cooley E, Silva EM, et al. The influence of inherent soil factors and agricultural management on soil organic matter. Ecosphere. 2023;14(3):. doi: 10.1002/ecs2.4459

[48] Donovan P. Measuring soil carbon change: a flexible, practical, local method. 2013.

